# Supplementary material for: A Fabry-Pérot cavity coupled surface plasmon photodiode for electrical biomolecular sensing
Source: Nat Commun. 2021 Nov 10;12:6483. doi: 10.1038/s41467-021-26652-7 (PMC8580965; doi:10.1038/s41467-021-26652-7)
Supplement: Supplementary file 1 — Supplementary Information [file 41467_2021_26652_MOESM1_ESM.pdf]

A Fabry-Pérot cavity coupled surface plasmon photodiode for electrical biomolecular sensing

Giles Allison, Amrita Kumar Sana, Yuta Ogawa, Hidemi Kato, Kosei Ueno, Hiroaki Misawa, Koki Hayashi, and Hironori Suzuki

Supplementary Note 1: Angle Conversion

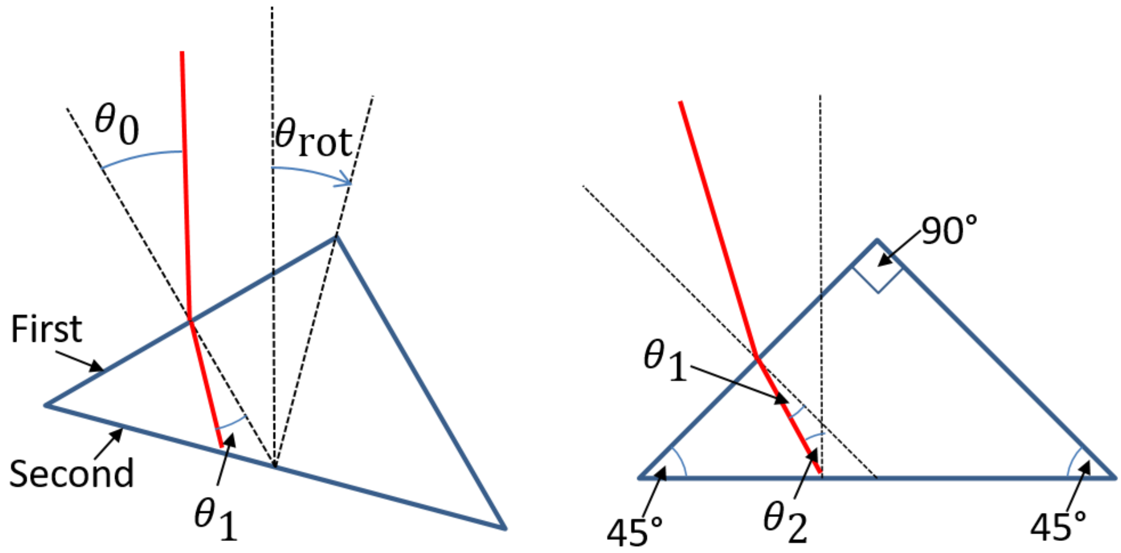

Supplementary Figure 1: Relationship between rotation angle and angle within layer structure.

A 90°/45°/45° prism was used throughout. With reference to Supplementary Figure 1, the angles within each layer can be converted from the experimental angle of rotation, θ_{rot}, using Snell's law and trigonometric identities with the following formulae.

$$\theta_0 = \theta_{\mathrm{rot}} - 45 \tag{1}$$

$$n_{\mathrm{air}} \sin\theta_0 = n_{\mathrm{glass}} \sin\theta_1 \tag{2}$$

$$\theta_2 = (180 - 90 - 45) - \theta_1 \tag{3}$$

where θ_0, θ_1, and θ_2 are respectively the angle in air with respect to the first surface of the prism, angle in glass with respect to the first surface of the prism, and angle in glass with respect to the second surface of the prism. n_{air} and n_{glass} are the refractive index of air and glass respectively. Furthermore, the angle, θ_X, within layer X (ITO, Si, or TiO_2) with respect to the normal of the layer is given by

$$n_X \sin\theta_X = n_{\mathrm{glass}} \sin\theta_2 \tag{4}$$

where n_X is the refractive index of layer X.

Supplementary Note 2: Silicon Transmittance

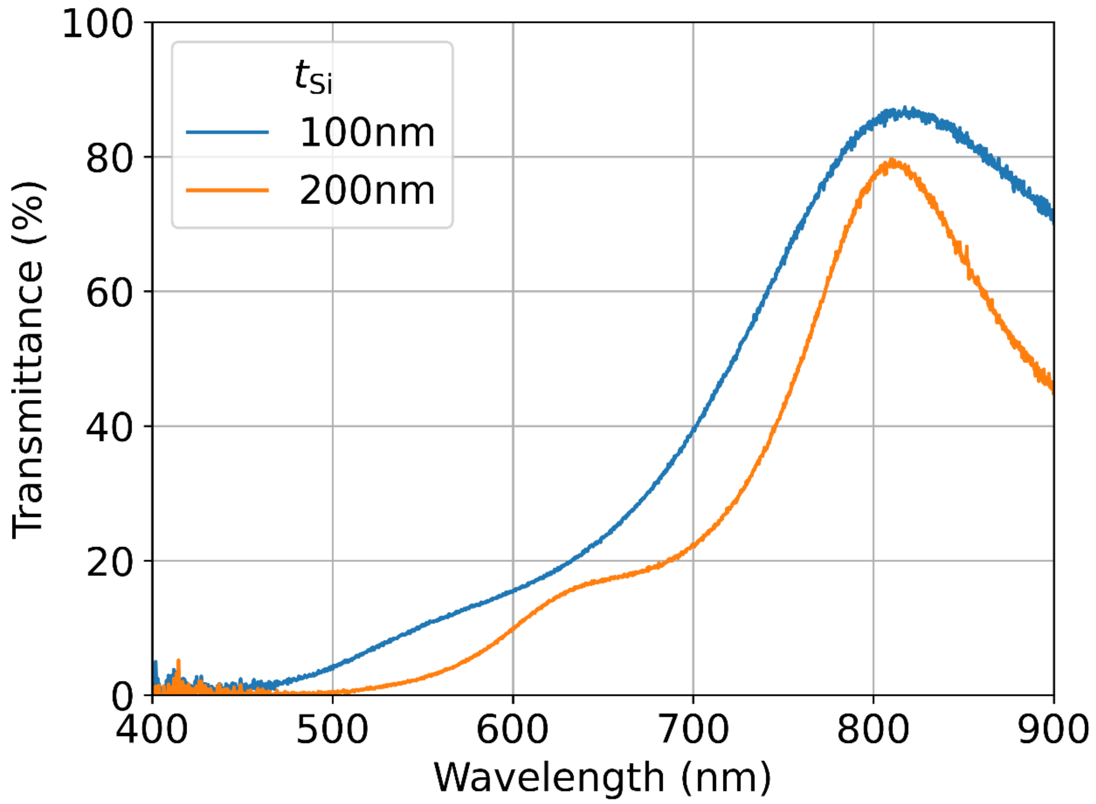

Supplementary Figure 2: Transmittance of amorphous silicon with thickness 100 nm (blue) and 200 nm (orange). Source data are provided as a Source Data file.

Supplementary Note 3: Cavity modes

Fabry-Pérot resonances are observed in the plot of electric field intensity (Supplementary Figure 3). For all silicon thicknesses and wavelengths the electric field is angle dependent due to the formation of a surface plasmon as described in the main text. In addition, periodic oscillations of the electric field intensity, $|\mathbf{E}|^2$, are seen as a function of distance through the silicon layer. The periodicity is dependent on the wavelength and refractive index of the silicon layer. A cavity mode is formed when the silicon thickness is equal to one of these oscillations (as is the case for the first, third, fifth, and seventh plots). As a result of Fabry-Pérot resonance, $|\mathbf{E}|^2$ is enhanced at these silicon thicknesses compared to non-resonant thickness (second, fourth, and sixth plots).

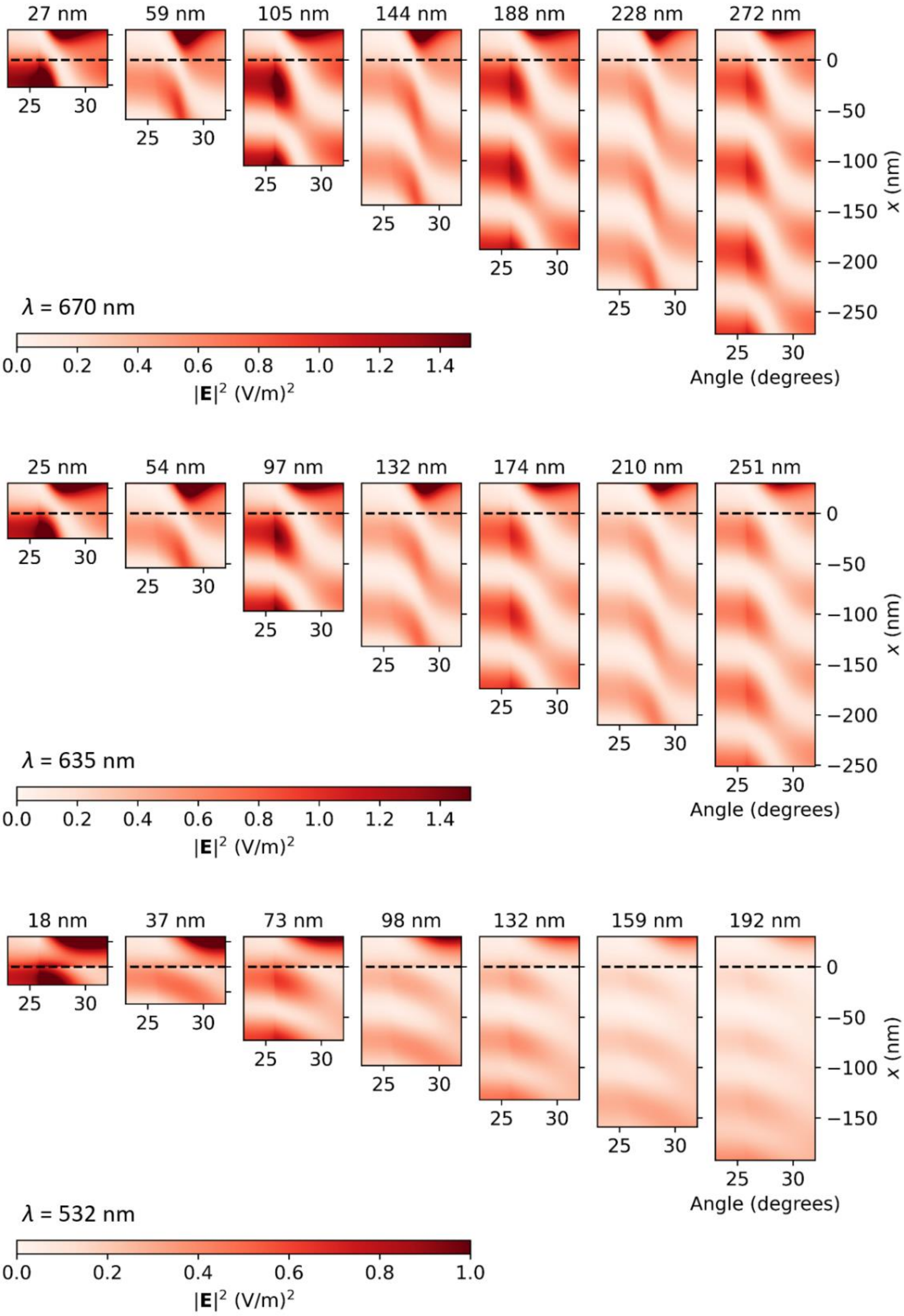

Supplementary Figure 3: Cavity Modes. Electric field intensity, $|\mathbf{E}|^2$, for different silicon thickness and three different laser wavelengths. Dashed lines indicate the interface between silicon and gold. For each wavelength, the first, third, fifth, and seventh silicon thicknesses show larger $|\mathbf{E}|^2$ than the second, fourth, and sixth due to the formation of Fabry-Perot resonances. The gold layer has thickness of 30 nm in all plots.

Supplementary Note 4: Electric field intensity and Contributing Absorptance of the TiO_2-based device

Supplementary Figure 4a shows the electric field intensity in of the TiO_2 based device as a function of angle and distance through the structure. The TiO_2 layer thickness is 200 nm and Au layer thickness is 50 nm. The data closely resembles that of the silicon based device shown in Fig. 1d of the main text. The difference between the two types of structure is that TiO_2 is optically transparent, so no electrons are generated in this layer directly. Instead, electrons can only be generated within the gold layer and then emitted into the TiO_2 layer. Considering first the non-resonant angles, (θ<29° and θ>31°), the electric field intensity is greatest at x ~ 150 nm and this region of high intensity penetrates into the Au layer (up to x ~ 210 nm). The majority of electron photogeneration therefore occurs near the TiO_2/Au interface resulting in large emission into the semiconductor and large photocurrent. In contrast, at the resonant angle, θ_{SPR} ~ 30°, the electric field intensity is highest near the Au/Air interface and the intensity is reduced near the TiO_2/Au interface. As a result, the photocurrent decreases at the SPR angle despite the much larger absorption. To simulate the photocurrent dependence we introduce the empirical quantity “Contributing Absorptance” as defined in the Methods section of the main text. By limiting the integration of the electric field intensity in the gold layer to only the 10 nm closest to the TiO_2/Au interface, i.e. between 200 and 210 nm, we are in good agreement with the experimental photocurrent (Supplementary Figure 4b).

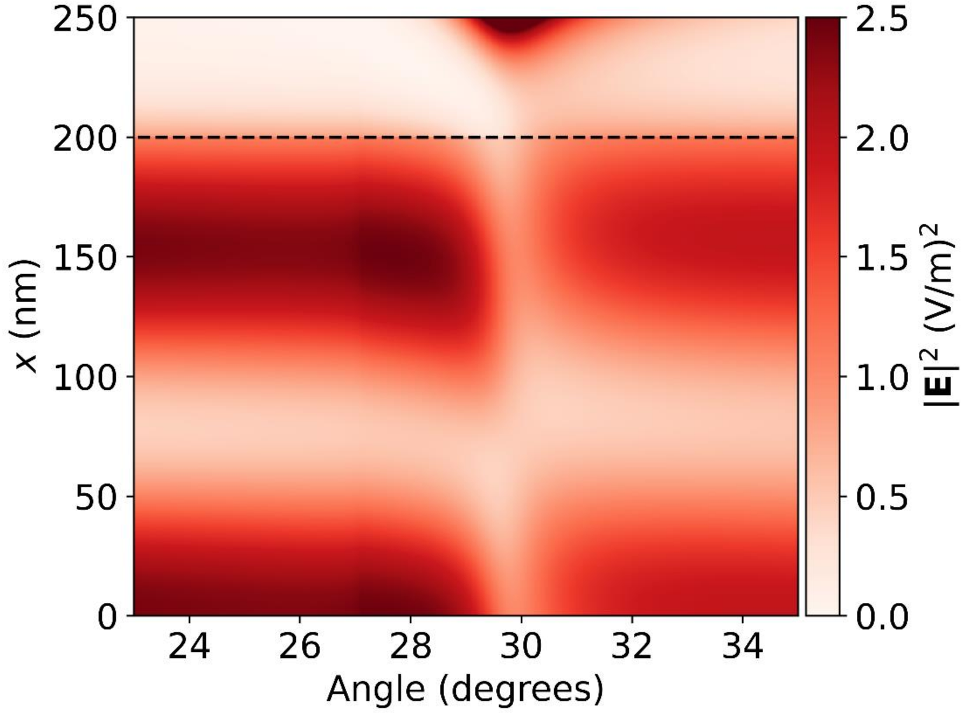

Supplementary Figure 4a: Angular and spatial dependence of the electric field intensity of the TiO_2-based device. Dashed line indicates the interface between TiO_2 and gold.

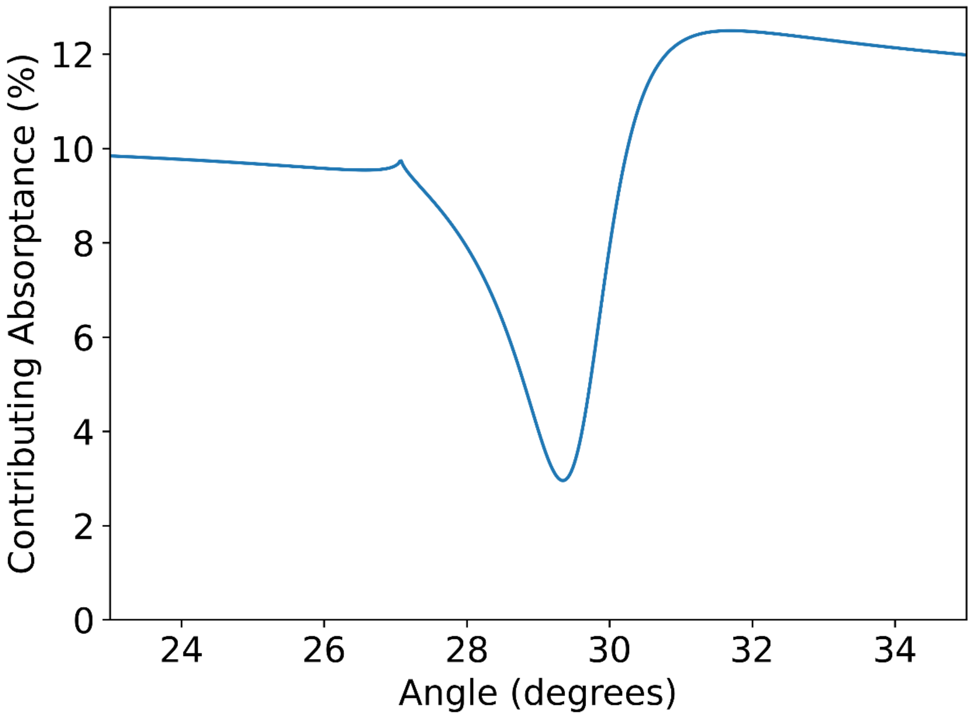

Supplementary Figure 4b: Contributing Absorptance of the TiO_2-based device.

Supplementary Note 5: Antibody sensing using BLItz

To benchmark the performance of our biosensor we compare its performance with that of the commonly used biosensor, BLItz. It should be noted that the two types of biosensor operate using entirely different principles. Our sensor operates on the principle of surface plasmon resonance whereas the BLItz sensor operates on the principle of bio-layer interferometry. In Supplementary Figure 5 in an automatically timed process using a BLItz, we perform exactly the same procedure with the same antibodies and antigens as in Figure 4 of the main text. The antigen-antibody interaction between the two proteins is detected as a change of binding in units of nm. In Fig. Supplementary Figure **5a** the raw binding data is offset using the same procedure as in the main text. The interaction increases logarithmically with time and is analogous to the change of photocurrent plotted in Figure **4a** of the main text. However, unlike the change of photocurrent of our sensor, the change of binding shows no difference for low concentrations of antigen below 1.0 nM. Furthermore, the fit to a logistic function of the change of binding after 1 minute plotted in Supplementary Figure **5b** indicates saturation at both low and high concentrations. The saturation at low concentrations suggests that our SPR-based biosensor outperforms the BLItz by more than an order of magnitude.

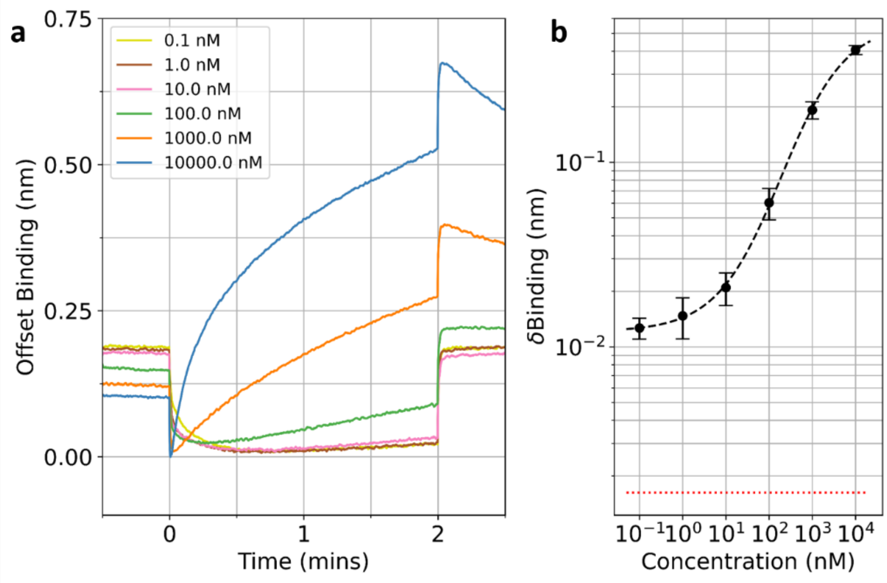

Supplementary Figure 5: Detection of MBP-SARS-CoV-2 Nucleocapsid protein using BLItz. a Binding as a function of time. The sensor is initially covered with a buffer solution. At time = 0, the buffer solution is removed and replaced with solutions containing different concentrations of antigen (MBP-SARS-CoV-2NC). At time = 2 mins the antigen solution is replaced with buffer solution. No difference in binding is seen between concentrations of 1.0 and 0.1 nM. **b** Change of binding after 1 min as a function of antigen concentration. Symbols and error bars are the mean and SD of three different sensors. The dotted red line is the noise floor of the sensor as defined by the standard deviation of the binding during the initial buffer stage (time = -0.5 to 0 mins). The dashed black line is a best-fit logistic function guide to the eye that indicates

saturation of the sensor at both low and high concentration. Source data are provided as a Source Data file.

Supplementary Note 6: Distinguishing between changes of signal due to bulk and interaction effects

Our device detects changes of refractive index in the solution above the gold surface. Such changes of RI may be due to the bulk properties of the solution (as in the glycerol experiments in Fig. 3 of the main text) or localised properties due to antigen-antibody interactions (as in Fig. 4 of the main text). Supplementary Figure 6 **a-c** illustrates the difference between these two situations. The initial uniform RI is shown in **a**. The bulk effect is shown in **b** as a change to another uniform RI. The localised effect is shown in **c** in which the RI only changes near the surface of the gold layer. In our experiment the antigen-antibody interactions are localised within the carboxymethyl dextran layer over a typical length scale of several 100 nm.

In Supplementary Figure 6 **d-g** we see the result of this difference between bulk and localised effects using plots of the simulated reflection of a traditional SPR device (a 50nm gold film on a glass substrate) and the contributing absorptance of the silicon-based device introduced in this work. The blue lines indicate the initial R and CA for a uniform solution with RI = 1.333. The orange lines illustrate the bulk effect by changing the uniform solution to RI = 1.35. The green lines illustrate the interaction effect by splitting the solution into two regions as indicated in **c**; in the upper portion of the solution (more than 200 nm above the gold surface) the RI remains the initial 1.333, and in the lower portion of the solution (up to 200 nm above the gold surface) RI = 1.365. [The changes of RI used in this figure are much larger than those in a typical experiment in order to clearly illustrate the differences between bulk and interaction effects.]

The bulk effect is seen as changes of both the total internal reflection (θ_{TIR}) angle and the angle of minimum signal (θ_{SPR}). On the other hand interaction effects only change the angle of minimum reflection. The angle sweep mode of operation requires measurement of both θ_{TIR} and θ_{SPR} to ensure that changes are due to antigen-antibody interactions and not inconsistencies of the RI as the buffer solution is exchanged with the antigen containing solution. On the other hand, it is clear from the preceding experiments using the BLItz that antibody-antigen interactions have an interaction time constant of the order of 1 minute as opposed to bulk effects that are effectively instantaneous. In the fixed angle mode of operation (**f** and **g**) we take advantage of this time dependence and require only a single fit to detect antibody-antigen interactions.

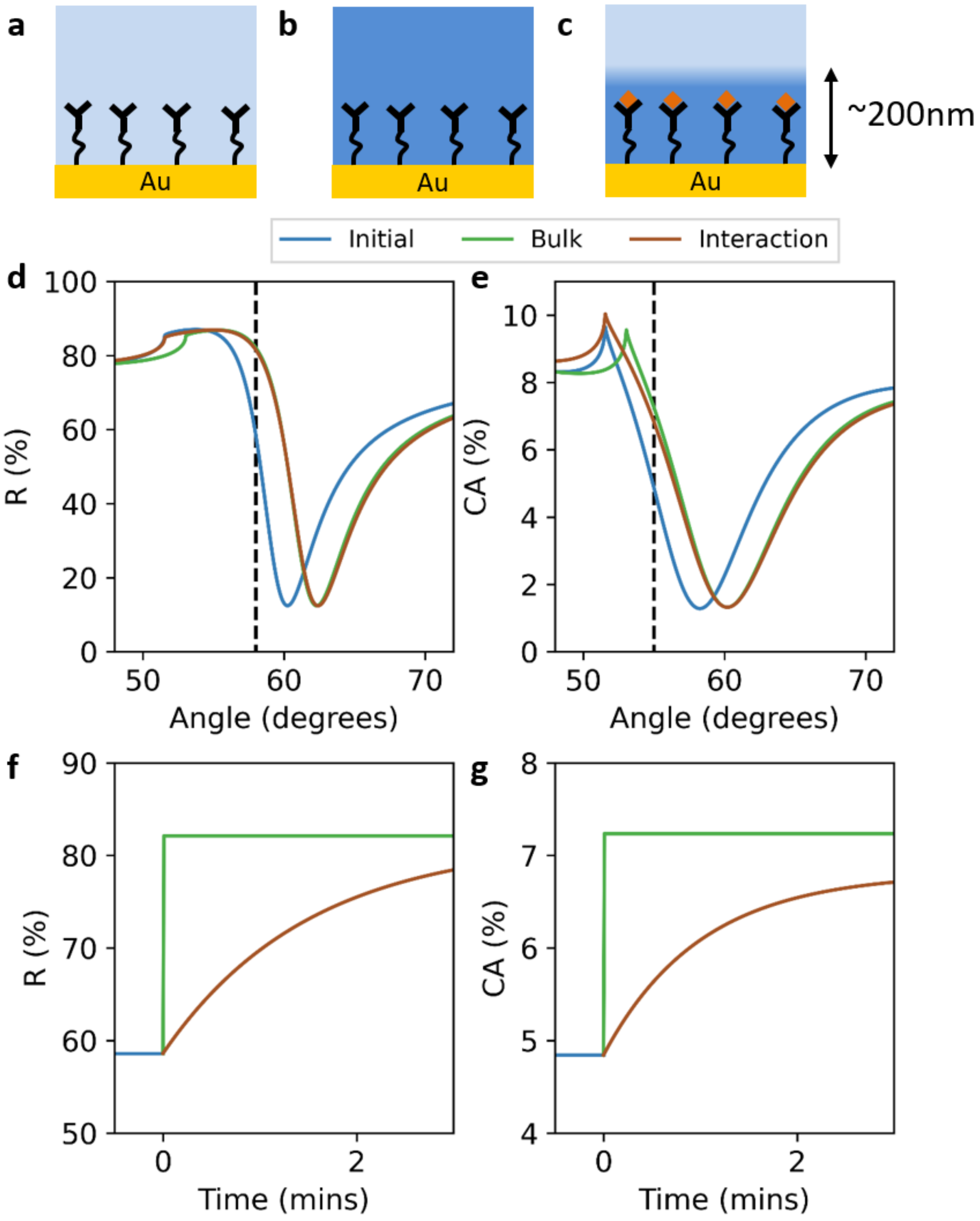

Supplementary Figure 6: Simulated change of reflectance and CA due to bulk changes of RI and antigen-antibody interactions. Sketches **a**-**c** indicate bulk and localised changes of RI as described in the text. Figures **d** and **f** are for a conventional SPR device. Figures **e** and **g** are for the silicon-based device introduced in this work. **f** and **g** are model time dependences at the angles indicated in **d** and **e** using a time constant of 60s.
