# Supplementary material for: A Fabry-Pérot cavity coupled surface plasmon photodiode for electrical biomolecular sensing
Source: Nat Commun. 2021 Nov 10;12:6483. doi: 10.1038/s41467-021-26652-7 (PMC8580965; doi:10.1038/s41467-021-26652-7)
Supplement: Supplementary file 3 — Description of Additional Supplementary Files [file 41467_2021_26652_MOESM3_ESM.docx]

**Description of Additional Supplementary Files**

**Supplementary Movie**

The panel covering the sensor chip is opened and the sensor chip is covered with solution. Upon pressing the measurement button the photocurrent is recorded as a function of time. The initial zero-value dark current is followed by a step-like increase upon illumination and a final zero-value dark current. A clear difference is observed between the photocurrent level when the sensor chip is covered with 20% glycerol (blue line) and when the sensor chip is covered by water (the prerecorded yellow line). The prototype model uses the Si-based chip represented in Fig. 1 of the main text and also contains the laser source, optical components (including prism), sensor chip, current amplifier, control unit and battery. The prototype model is connected to the computer laptop via a USB interface to record and display the data.
